# Tumors in General, and Hard Fibrous Tumors

**Published:** 1860-03

**Authors:** Titus Deville

**Affiliations:** Professor of Anatomy in Medical Department of Lind University


THE CHICAGO
### MEDICAL EXAMINER

VOL. I. MARCH, 1860. No. 3

### ORIGINAL COMMUNICATIONS.

### ON TUMORS IN GENERAL AND HARD FIBROUS TUMORS.

By TITUS DEVILLE, M. D.,
Professor of Anatomy in Medical Department of Lind University.

(Read to the Chicago Medical Society.)

I.—*Origin of Tumors. Theory of development and growth.*

A.—The elementary textures are of the same kind and produced in the same way as they are in normal tissues, external, fatty, glandular and vascular tumors.

B.—The blood furnishes a blastema in which cells are developed, and which undergo the same metamorphosis observed in the development of similar textures in the embryo, as in fibrous and osseous tumors.

C.—By multiplication of cells, which either already exist or are formed, and by their numbers and mode of arrangement alter the normal relations of the textures, as in epithelial, cartilaginous and cancerous growths.

D.—One or more of these modes of development and growth may be combined, and thus we get fibro-cartilaginous growths, etc.

It has been asserted that tumors have their origin from extravasated blood or *inflammatory* exudation becoming organized, but this is probably incorrect.

1st.—Because when these materials organize, they gradually *decrease*, and become more like the surrounding parts, whilst tumors gradually *increase*, and pursue a development and growth peculiar to them. In the inflammatory exudation there is a continuous tendency *towards conformity* with the type of the body; in tumors a *direct and continuous deviation* from it in shape and volume, if not in texture.

2d.—A tumor may be like the natural tissues of the body, but as a growing substance it is unlike them, because it grows out of proportion to the rest, and apparently with inherent power and with no seeming purpose.

3d.—Even when a tumor has attained its full growth, excepting in the rare case where a tumor is absorbed, there is no indication of a return to the normal type or condition of the body, we find no improvement as in the case of organized lymph, exuded in inflammation, nor adaptation to purpose; no assumption of a more natural shape, but has rather a tendency to degenerate. From these premises we demonstrate the nearly independent nature of their growth and cessation, and whilst forming part of the bodily organism and borrowing from it the materials to nourish them, tumors grow, maintain themselves or degenerate, by virtue of forces inherent in themselves.

II.—*Elementary Structure of Tumors.*

1st.—Molecules and Granules.

2d.—Nuclei.

3d.—Cells.

4th.—Fibres.

5th.—Tubes (particularly Vascular ones.)

6th.—Crystals, or irregular masses of mineral matter.

These elements, though severally combined in tumors, do not serve specially to characterize any particular one, for the reason that tumors very unlike each other in their external characters and natures, may be composed of the same elements; external, cystic, glandular, cartilaginous and cancerous growths, are all fibro-cellular.

III.—*Formation of Fibrous Growths.* Two kinds.

1st.—By an exudation in which new fibres are formed.

2d.—By hypertrophy of fibrous tissue already existing, by their division or enlargement, external hypertrophy of voluntary and involuntary muscular fibres.

The first form presents us with several varieties:

A.—*Molecular Fibres.* By the coagulation of the exudation in which the molecules arrange themselves in a linear manner, as in buffy coat of a coagulum.

B.—*Nuclear Fibres.* By nuclei being formed in exudation, which elongate and shew a tendency to fibrillate.

C.—*Cell Fibres.* By cells being formed which become fusiform, split up, and form fibres, as in formation of healthy tissues.

In these three ways we get all varieties and forms of fibrous element, from the most delicate areolar tissue to structures as dense as ligament or fibro-cartilage.

IV.—*Pathological Fibrous Tissue is met with :*

1st.—In form of cicatrix.

2d.—In white patches on pericardium, pleura, and peritoneum, the consequence of chronic exudation on serous membranes.

3d.—Thickening after sub-cutaneous section of tendons.

4th.—Indurations and thickening of skin, cirrhosis of liver, lung and kidney from an increase of the areolar tissue.

5th. Tumors which are divisible into

A.—Sarcomatous or *soft* fibrous tumors.

B.—Dermoid or *hard* fibrous tumors.

C.—Neuromatous fibrous tumors.

V. — *General character of Dermoid or hard Fibrous Tumors.**

They have been called dermoid from their resemblance to dermis, and if we take the dermis of some large mammal, as the whale, the analogy of structure is very evident. Their color is generally white, but sometimes inclining to yellow ; tough, heavy, and of considerable density, which varies from that of a tendon to that of ligament and fibro-cartilage. To the touch fibrous tumors are usually very firm, often extremely so, they may even be as hard and incompressible as hard cancers ; if they are soft or fleshy, or succulent, it is from œdema or inflammatory softness, or infiltration of their substance. Fibrous tumors seem to grow in a spherical or oval

* Dr. Baillie described fibrous tumors, or fibrous bodies, as "hard fleshy tubercle" of uterus. Cruveilhier calls them "productions fibreuses parasitaires." The older pathologists called them "schirrus," with which they were confounded. Wm. Hunter was the first to point out their true nature, and to separate them from the classification of tubercular and schirrous tumors. But it is principally at the commencement of the present century that their true nature has been studied and ably commented upon by Dupuytren, Laennec, Cruveilhier and Andral.

shape, the surface being smooth or superficially lobed, but we find them differ occasionally from the type when they mutually press on one another, or are pressed upon by surrounding parts. When a fibrous tumor is pendulous, the lowest part grows most, or is most swollen, and thus it assumes a pyriform shape rather than a spheroidal, but retains its smooth surface; when it grows into a cavity it is apt to assume the shape of that cavity, or else becomes deeply lobed. They are so elastic, that when cut their surfaces rise in convexities like those of the intervertebral substance, presenting numerous white glistening fibres, intimately interwoven together. The bundles of fibres are often arranged in concentric circles around many centres, so that in the section we have a vague imitation of the aspect of the intervertebral substance. On making a section of this large fibrous tumor of the uterus, a number of these concentric masses are found to exist, and the fibres are seen coursing and curling in all directions, but more or less disposed to include certain definite spaces within them, so that we see several small round circumscribed tumors imbedded in the walls of the uterus, many of them being coated with a layer of calcareous matter. Now, as these grow, they project from the surface, and then slowly enlarge within the cavity of abdomen. Very often a bunch of them grows from the surface, as seen in the specimen under consideration, forming a mass of distinct tumors. Occasionally they become pedunculated, and some of them may here be seen hanging by a slender cord to the fundus, and they are occasionally found loose in the abdomen from rupture of the pedicle. They are not very vascular, though there is some difference in this respect, some approaching the pinkish hue and softness of sarcomatous growths; others, like this tumor, being of a dead white; of extreme density, and but few vessels. They become more softened and vascular during pregnancy, assuming a bluish-red color, but as soon as the uterus returns to its natural shape, the morbid growth also resumes its ordinary character. We may state generally that fibrous tumors are modified in their character and structure towards an imitation of the tissues in or near which they are severally placed.

From the all pervading character of fibrous and connective tissue, it will not be a matter of surprise to find that these tumors are of most frequent occurrence in every part, but by far their most common seat is the uterus ; again, that of all the abnormal formations which occur in the uterus, fibrous tumors are the most common. According to Bayle's calculations, they are found in twenty per cent. of the women who die after 35 years of age. Fibrous tumors may occur near, as well as in the uterus ; in those parts in which smooth muscular tissue, like that of the uterus is found to extend, viz : Utero-rectal, and utero-vesical folds and the broad ligaments.

The uterus consists of muscular fibre, but which is scarcely distinguishable by an unpractised eye from simple fibrous tissue in the unimpregnated state, and the tumors which grow within it are composed of the same elements, (i. e. smooth or organic muscular fibres are more or less abundant, like as in the uterus itself.) It is, however, when the organ is rapidly growing in size, during pregnancy, that new well formed muscular fibres become developed, and then it is that a tumor, if one be present, also increases, in accordance with the activity of the nutrition of the uterus, the muscular nature of the tumor becomes apparent, and in consequence of this, the name '*muscular tumor*' has been substituted by Vogel for that of fibrous. We cannot say that they are strictly muscular tumors, but the *mingling* of muscular fibres, in imitation of the tissue of the uterus, is constant in these tumors. It is observed that when tumors are developed in the substance of the organ, its muscular fibres become hypertrophied as in pregnancy; its blood vessels enlarged, and venous sinuses may, as in the condition of pregnancy, give rise to the 'bruit de souffle.' (Preparations 375 to 377. M. D.) Very generally we find elastic fibres intermingled with the more abundant fibrous tissue.

Fibrous tumors growing in solid organs have usually a complete fibro-cellular capsule, and in the uterine walls it is peculiarly dry and loose, so that when we cut on the tumor it almost of itself escapes from its cavity. The number of these tumors varies, rarely unique, more commonly mutiple and isolated. M. Huguier has given a case in which these mutiple tumors were enclosed by one common enveloping membrane, constituted by a layer of hypertrophied areolar tissue. Their volume varies from a grain of flax seed to an adult head, and even larger. Their growth is through nucleated blastema. Growth generally slow and painless, so slow that tumors of thirty or more years existence are found still far short of the enormous dimensions which they ordinarily attain.

No general rule can be given, especially as the rate of growth is influenced by the resistance afforded by the more or less yielding parts around. The extent of growth seems unlimited; amongst fibrous tumors are found the heaviest yet known, 50, 60, and 70 lbs. Walter wrote his essay on a tumor which weighed 71 lbs., and refers to one which weighed 74 lbs. My friend Dr. Francis, of New York, described one in the *American Journal of Medical Sciences*, over 100 lbs. Cancer cells are never found in them; in cases in which they were said to have existed, it was *merely the fibrous tissue around the cancer which was hypertrophied*, and evidently was not a tumor in a degenerate condition. Occasionally, after extirpation, they have been found to recur and pursue *a course like cancer*, growing in internal organs, with a tendency to sloughing or ulceration, involving adjacent structures. (*Vide* cases reported by Paget and Syme.) Hence they have been termed 'malignant fibrous tumors;' but the question of real malignancy of structure is *doubtful*, inasmuch as they are identical in all respects of structure and chemical composition with the fibrous tumors of the uterus, excepting their muscular fibres.. The probable fact is, that in such cases the surgeon really leaves multitudes of germs behind, infiltrated among muscles and neighboring parts, which may be detected there by the microscope, although invisible to the naked eye, and thus continue to propagate the disease. The principal character by which fibrous tumors differ from those of cancer, is, that on scraping them, *no milky juice* is exuded. The firmness of these tumors, or hardness when cut with a knife, is sufficient to characterize them, and especially if there be any gritty or earthy matter within them, which never occurs in softer or cancerous growths. When they approach the skin they may ulcerate, and have sometimes been mistaken for a cancerous degeneration. In order to prevent confusion in the classification, I prefer to call them uterine fibrous tumors, which grow internally, under the name of '*internal and pedunculated fibrous tumors.*' They are continuous out-growths of and from the substance of the uterus, the membranous, muscular and fibrous tissues of the uterus, growing in various proportions into its cavity and that of the vagina. The fibrous tumor polypus, is sometimes called '*muscular polypus*,' from the great development of muscular fibres, which would appear to be an effort of nature to rid itself of these tumors by expelling them from the cavity.

VI.—*Special characters, etc., of hard Uterine Fibrous Tumors.*

1st.—*Their seat is different* from the soft polypi, as well as the structure. The hard fibrous polypi are nearly always attached to the *fundus*, or on one of the walls of uterus (well shewn in specimens Nos. 366, 368, 369 Musee Dupuytren); whilst the soft polypi are situated in the *neck* or its immediate neighborhood; and which are nothing more than an hypertrophied condition of the mucous and glandular structures of this region.

M. Huguier has given a remarkable exceptional case of a fibrous tumor of the neck of the uterus, which had caused an elongation of its posterior lip.

2d.—*The disposition of the fibres* is different from the soft polypi; in the hard polypi the fibrous tissue is in *hard concentric layers*; on the contrary, in the *soft* the fibrous tissue *radiates*, and the bundles of fibres are separated by cavities which enclose ordinarily a clear viscid fluid, hence these have been called '*gelatinous polypi.*'—Preparations from 356 to 360 M. D.

3d.—The whole wall of the uterus may be crammed with fibrous tumors, whilst others project from it into the peritoneal cavity. It has been observed that when a fibrous tumor fills the cavity of the uterus, or projects from it into the vagina, it is not usual for another to be found in its walls. They do occur, but comparatively rare. It is yet much rarer for fibrous tumors to occur in any other part, when they do exist in the uterus. One *apparently exceptional case* is recorded by Dr. Sutherland, where a fibrous tumor was found in the groin of a lunatic 42 years old, *but if it were connected with the round ligament*, it might be an *example* of the rule.

4th.—Fibrous tumors may occupy all parts of the uterus, but their seat of predilection is in the following order: first, walls of uterus, principally posterior; second, Fundus; third, anterior wall of neck.

5th.—When the tumor is developed from *the mucous surface* of uterus (sub-mucous tissue) it soon becomes pedunculated, distends the cavity, and may attain such development as to simulate pregnancy, the neck and os dilate, and it may protrude into the vagina, simulating pregnancy arrived at its last stage.

6th.—When the pedicle is attached to fundus, it may turn the uterus inside out. (*Vide* preparation No. 377 Musee Dupuytren.) Such an accident would render it difficult in the operation of extirpation of the tumor to distinguish between the fibrous tumor and the wall of the uterus; and it is important not to mistake for the pedicle the invaginated portion of the uterus, because if cut the uterus *would then communicate with he peritoneal cavity.*

7th.—When this pedunculated tumor is developed from the sub-peritoneal tissue of the uterus, the volume which it acquires may be considerable, and it may distend the abdominal cavity by rising out of the pelvis, the uterus being much lengthened by being drawn upon by the pedicle, and the neck, as in the case of pregnancy, would be found higher.

8th.—According to Rokitansky the appearance of these tumors is never before the 30th year, and most frequently after the 40th.

9th.—Whilst fibrous tumors are most frequently generated from the fundus, carcinomatous disease is developed from the inferior segment of the uterus.

10th.—Those tumors which are embedded in the walls of the uterus appear to be encysted, being limited by an enveloping fibrous membrane, a sort of cyst. These tumors are but little vascular, we scarcely ever meet with vessels but on their surface in the cellular tissue, rarely do we see them penetrate into the centre of the tumor, if two or three fibrous tumors are united together by cellular tissue, we find vessels in the connective tissue. For these reasons the tumors themselves cannot readily inflame, but their enveloping fibrous membrane, which is very vascular, may become the seat of inflammation, which if it has been intense and existed some time may render the tumor more or less deeply red and soft.

11th.—One thing very remarkable is that these fibrous tumors, when they lie free in the abdominal cavity by rupture of the pedicle, they may remain a long time without undergoing any alteration, although they are exposed to putrefactive agencies, (i. e. Heat and moisture.) Some authors have supposed this absence of putrefaction to be due principally to the absence of the contact of air, and the slight vascularity of these tumors, and under this condition presenting an analogy to foreign bodies in the joints. We may consider heat as necessary to their life, at the same time they are surrounded by serous exhalations. It is possible that the heat favors their imbibition of these liquids, which may be a mode of vitality for them up to a certain point, but it is more probable that they undergo calcareous degeneration. Some doubt still exists amongst pathologists as to the existence of these tumors lying free in the abdominal cavity.

*Degeneration of fibrous tumors are of three kinds :* Calcification, cysts and softening or disintegration, thus giving rise to the terms Fibro-cystic and Fibro-calcareous tumors.

1st.—Calcareous matter is found to exist in these tumors in two forms, either coating them with a thin layer of chalky substance, or deposited abundantly throughout the tumor so that it forms a hard mass. If it grows inwards, the cavity of the uterus may be occupied by such a mass. The structure is amorphous, possessing none of the characters of true bone, and for this reason it cannot be supposed to be an exostosis, as was thought from a specimen found in a churchyard. An oval, coral-like mass, five inches long, was sent to J. Hunter, as a urinary calculus ; on analysis it was found to contain 18½ per cent. of animal matter, consisting ot gelatine, with a small proportion of albumen, and its other chief constituents were phosphate and carbonate of lime, the carbonate being greater than in human bone. Such masses consist of an amorphous and disorderly deposit of salts of lime and other bases in combination with fibrous tissue; and not true osseous tissue, which is rarely formed in fibrous tumors of the uterus. Though the degeneration of fibrous tumors into bone is denied by most pathologists, yet a specimen which I examined, taken from a subject in the Ecole Partique of Paris, there was a large osseous tumor of the uterus of several pounds weight. It was examined microscopically by M. Martin Magron and M. Robin, and was presented to the Societe Anatomique de Paris. Lebert (Physiologie pathologique, 1842, Tome 2me atlas fig. 22) states that on two occasions he has seen true bone produced in these tumors. Wedl has figured true bone in the interior of these growths. This process of calcification is important as being a manifestation of a loss of formative power in the tumor. When in this state they never grow, and are as inactive as the calcified arteries of old age.

2d—The formation of cysts is not unfrequent in fibrous tumors, especially when the textures are more than usually loose. It may be caused by a local softening and liquefaction of a part of the tumor, with effusion of fluid in the affected part, or to an accumulation of fluid in the interspaces of the intersecting bands, and these are the probable modes of formation of the roughly bounded cavities that may be found in uterine tumors. But in others, of small size, and highly smooth polished internal surfaces, it probably depends on a process of cyst formation, corresponding with that traced in cystic disease of breast and testes. They contain a serous or gelatinous fluid.

3rd.—Softening or disintegration is usually the result of inflammation of the hypertrophied sub-mucous or sub-peritoneal areolar tissue enveloping these tumors, and affecting their nutrition.

*Complications arising from Uterine Fibrous Tumors.*

A.—On uterus itself.

B.—On surrounding organs and tissues.

1st—The interstices of a fibrous tumor may become dilated into cysts or cavities containing a serous fluid from excessive exhalation of the intervening cellular tissue, and it is of extreme importance, inasmuch as they present fluctuation, and may be mistaken for ovarian dropsy, hydrometra, acephalocyst of the uterus, or pregnancy. Several cases have happened in which a cyst of large size, developed in a fibrous tumor of the uterus has been mistaken and treated for an ovarian cyst. There is a specimen in Museum of Royal College of Surgeons, in which Sir E. Home twice tapped. Another in the Museum of St. George's Hospital, in which M. Cesar Hawkins drew off fifteen pints from a cystic tumor developed in the side of the uterine wall.

2nd.—In the unimpregnated uterus all forms, but especially the sub-mucous and interstitial are apt to be accompanied by severe recurrent hemorrhage reducing the patient to the last stage of exhaustion, produced by hypertrophy, inflammation and even ulceration of the mucous membrane necessitating their extirpation.

3rd.—M. Barnetche gives an example of an abscess being developed in the thickness of one of these fibrous tumors, occurring in a woman who died in forty-eight hours after a difficult labor, it is probable that the tumor was multiple, and that the abcess was developed in the hypertrophied cellular tissue uniting them.

Dr. Robert Lee found in the centre of a fibrous tumor, a cyst filled by a clot of blood. The same explanation as in last. It was in all probability developed outside the tumor.

4th.—M. Chassignac, (Bull. Soc. anatomique de Paris, 1843, p. 10) gives a very rare example where a fibrous tumor caused an occlusion of the uterine cavity, by adhesion of the opposing surfaces of the mucous membrane.

B.—On surrounding organs and tissues.

1st.—One of the most common is inflammation of the sub-peritoneal tissue, exciting local peritonitis, which may result either in adhesion of the tumor to surrounding organs and walls of the cavity, or in softening abcess, etc. Occasionally the uretur has been seen to be enclosed within an organized band of areolar tissue, interfering with the function of the kidney of that side. (Such a case occurred to me whilst at the Ecole de Medicine de Tours, France.)

2nd.—Not uncommonly, when the tumor is very large we meet with obstructions to the caval and portal circulations, from pressure on the great veins, giving rise to dropsical effusions, varicose veins, etc.

3rd—Great distention of the colon, from pressure on the sigmoid flexure, if the tumor be of considerable dimensions and projects freely into abdominal cavity on its left side, giving rise to obstinate constipation.

4th.—From pressure on the viscera of the abdomen, the functions of digestion and assimilation may be seriously impaired.

The author acknowledges his indebtedness to the works of Paget and Bennett on tumors, and to the various works referred to in the text.

---

### PUERPERAL ECLAMPSIA.

By RALPH N. ISHAM, M. D., Prof. Surgical Anatomy, Lind University, Chicago.

(Read before the Chicago Medical Society, Sept., 1859.

In the 10th No., 1st Vol., of the *Chicago Medical Journal*, was published the first part of an article upon Puerperal Eclampsia, intended to be finished in some succeeding issue. So long a time has since intervened, that it would not seem improper in now offering the continuation of that article, to give a resume of the views suggested at that time.

After defining puerperal eclampsia, I adverted to the fact that albuminous urine is always present in puerperal convulsions, and discussed the question of its identity with Bright's disease, detailing the reasons put forth in support of, and in opposition to such theory; aiming myself at the conclusion that the former may exist without Bright's kidney being present (since autopsies of eclampsia in a minority of cases, shewed the absence of Bright's disease) and arriving generally at the following results.

1st.—That equally in Bright's disease, and puerperal eclampsia, the presence of albumen in the urine and urea in the blood were due to a disordered state of the kidneys.

2d.—That while in Bright's disease, the results were due to a permanent structural disorganization, typical of that disease, in puerperal eclampsia many cases were due to a temporary functional derangement of the kidneys consequent upon a variety of causes, then enumerated.

3d.—That such temporary derangement might proceed so far as to cause Bright's disease.

4th.—That sometimes it does not, because the existing cause being removed by parturition the kidneys perform their functions normally.

5th.—That eclampsia occasionally occurring in Bright's disease are due to a special cause—the derangement of the kidneys, and consequent inability to eliminate the urea.

6th.—That the eclampsia is not due to the presence of the urea in the blood only, but is due to the change of the urea into carbonate of ammonia.

7th.—That the cause of this change is not known.

In this paper which I offer to the Society to night, I simply propose to state some of the experiments which have justified the theory that urea is the cause of eclampsia, and the manner in which this poison acts upon the system.

It is stated that urea may be injected into the veins of animals without serious results, and that even the extirpation of the kidneys will not give rise to uraemic intoxication, and that no urea is detected in the blood after this mutilation.

Thus Bichat injected filtered urine, and Bernard, Lehman, and Frerichs, have injected urea into the veins of animals without any evil consequences, so that it would appear that urea is one of the constituents of the circulation, and in fact it has been repeatedly detected of late by several observers, in the healthy blood; but the difficulty of so doing, is apparent, when it is remembered that the healthy kidneys remove it with such rapidity that it never exceeds the one-fiftieth of one per cent. of the circulation; but on the contrary, in carefully observed experiments on animals, from which the kidneys have been removed, and shown no symptoms of uremia, *vomiting appears*, and in the vomited matters urea is present. And in the subsequent experiments of Bernard and Barreswell, it appeared to them as a singular circumstance that in those animals which survived a length of time the removal of the kidneys, that a period varying from 24 to 48 hours elapsed before urea could be detected in the blood, and it became a question in what way the urea escaped from the system during this interval.

Two of the dogs died shortly after the *kidneys were removed*, the one from suffocation, the other from peritonitis. In the blood, which was carefully tested, no urea could be found, but the gastric fluids liberated on the addition of caustic-potash a suffocating ammoniacal odor, and the intestinal fluid and bile also disengaged large quantities of ammonia when similarly treated. They observed that in those animals which survived longer and died from exhaustion, that the gastric fluid was *increased* in quantity, and secreted during fasting and digestion; and that no urea could be detected in the blood until the animal became weak, and the gastric fluid diminished in consequence. Inferring from these experiments that after extirpation of the kidneys, the urea is eliminated in the form of ammonia by the intestinal tube, and chiefly by the gastric juice; that it is not to be detected in the blood until the vital powers have become so oppressed that the intestinal fluids are diminished in quantity to the extent that the supplementary channels for its separation are cut off.

A case recorded by Dr. McDowell, in *Dublin Hospital Gazette*, for March, 1856, is confirmatory of these facts.

A patient, aged 38, received a severe injury in the right side, followed by hæmaturia. In a few days vomiting supervened without symptoms of gastritis; tongue natural, and *no* tenderness of epigastrium. During the vomiting spells, the amount of blood discharged by the kidneys was greater, and the urinous smell very faint; whilst at other times it was strongly marked by the presence of ammonia. The erethism of the stomach increased, everything was rejected, and medicines useless; the mental faculties were unimpaired to the time death occurred.

An analysis of the vomited matters confirmed a suspicion of its containing both urea and ammonia; of the former in the proportion of one-fifth of a grain to fluid ounce. The renal secretions was alkaline. These results shew that the *stomach* assists the kidneys in removing urea in the form of ammonia (nitrogen) out of the system when those organs are in a diseased state, unable sufficiently to perform; and this vicarious action may explain the reason why in *all* cases of suppression uraemia does not take place. Although the given amount of vomited matters may contain less quantity of urea than the urine in a healthy condition, it is compensated for by the greater amount.

In the experiments of Bernard and Barreswell, the animals continued awake so long as this ammoniacal secretion poured out by the stomach continued, but directly it ceased, the symptoms of uraemic intoxication set in.

Frerichs' has repeated these experiments, but differs with Bernard and Barreswell in supposing that the resolution of urea into carb. of ammonia in this uraemic vomiting takes place in the stomach, by asserting that it is effected in the blood and vascular system. Now, to produce uraemic intoxication, we must have, first, urea accumulated in the blood; and secondly, it must be changed into carb. of ammonia.

Frerichs likens individuals whose blood is impregnated with urea, to animals into the veins of which amygdalin has been injected; from the presence of this agent alone, as of urea, they suffer little inconvenience, but a single sweet almond taken into the stomach suffices to deluge the blood with prussic acid, causing death instantly. The same reaction takes place through the stomach or its circulation as when an emulsion of sweet almonds is added to amygdaline, giving rise to a volatile oil and hydrocyanic acid, the emulsion acting the part of a ferment.

To prove the theory that eclampsia is the result of uræmia, it must be shewn

1st.—That in all cases of uraemic intoxication the urea must be converted into carb. of ammonia.

2d.—That the symptoms of *eclampsia can be produced by the presence of carb. of ammonia in the circulation.*

Two series of experiments by Frerich's are offered as the required proof.

In the first, a solution of 30 grs. of urea was injected into the veins of animals, from which the *kidneys had been previously removed.* In from 1¼ to 8 hours they became restless, vomited acid chyme, or alkaline, according to the state of fullness of the stomach at the commencement of the experiment. At the same time that ammonia was perceptible in the expired air, convulsions supervened, which occasionally ceased and returned again, and gradually passed into stupor with stertorous breathing.

In some cases convulsions were absent, and then sopor and coma were the first symptoms. After death, which occurred in from 2½ to 19 hours, ammonia was found in large quantities in the *blood.* The contents of the stomach remitted a strong urinous odor, and contained much ammonia; in *one* case only was it acid, and even then it contained ammonia. The stomach was injected, the brain and membranes being normal in appearance.

In the second series of experiments, a solution of carb. of ammonia was injected into the veins of animals; violent convulsions *instantly* ensued, and stupor quickly supervened; respiration was difficult, the expired breath was loaded with ammonia during the whole time. Gradually the latter disappeared, and by degrees the animals recovered their senses. When more carb. of ammonia was injected while the animals was in a state of stupor, the convulsions and vomiting recurred, and the urine and stools were passed involuntarily. After a lapse of 5 or 6 hours the ammonia again disappeared from the blood and the animal became again lively.

If the urea in the blood be too suddenly decomposed the symptoms are those of convulsions and apoplexy, but if the change takes place more slowly, the symptoms are those of typhus.

Thus it is said that in some blood diseases this element of ferment is seldom absent, as in scarlatina and typhus. The altered condition of the blood, which will not coagulate, the subsultus tendinum, and the presence of ammonia in those cases where a scanty secretion of urine is effected, being the symptoms.

In cases of scarlatina, followed by anasarca, the patients sometimes are affected by coma and convulsions, accompanied with albuminuria, and recovery often-times takes place without any organic change in the kidney following.*

If the patient, during the period of desquamation be exposed to cold, anasarca is produced. The kidneys in such cases are increased in size to 8 or 10 ozs., the outer coat peels off readily, the cortical substance is injected, giving rise to hemorrhage and exudation from rupture of the capillaries. The minute vessels and tubules become in part obliterated and in part compressed, and finally albumen is separated with the urine, and the imperfect action of the kidney prevents the elimination of urea.

The presence of ammonia and the very unfrequent occurrence of convulsions, look towards the fact, that the urea is passed off by the skin and mucous membranes in the form of ammonia; but as the vital powers sink from exhaustion in such cases, the patients die comatose, and sometimes with eclampsia. So also in typhus fever and cholera has urea and ammonia been found to be present, accompanied with symptoms of uræmia, in cases complicated with more or less suppression of urine; and many observers already regard the symptoms of what is termed cholera typhus as the result of uræmic intoxication.

